# Effect of rehabilitation length of stay on outcomes in individuals with traumatic brain injury or spinal cord injury: a systematic review protocol

**DOI:** 10.1186/2046-4053-2-59

**Published:** 2013-07-20

**Authors:** Marie-Eve Lamontagne, Cynthia Gagnon, Anne-Sophie Allaire, Luc Noreau

**Affiliations:** 1 Center for Interdisciplinary Research in Rehabilitation and Social Integration, Institut de réadaptation en déficience physique de Québec, 525, boul. Wilfrid-Hamel, Québec, QC, G1M 2S8, Canada; 2 Groupe de recherche interdisciplinaire sur les maladies neuromusculaires (GRIMN), Neuromuscular Clinic, Centre de réadaptation en déficience physique de Jonquière, 2230, rue de l’Hôpital, C. P. 1200, Jonquière, QC, G7X 7X2, Canada

**Keywords:** Brain injury, Spinal cord injury, Length of stay, Rehabilitation, Systematic review

## Abstract

**Background:**

Rehabilitation interventions are a key component of the services required by individuals with neurotrauma to recover or compensate for altered abilities and achieve optimal social participation. Primary studies have produced evidence of the effect of rehabilitation length of stay on individuals with neurotrauma. However, to date no systematic review of this evidence has been performed. This makes it difficult for managers and clinicians to base their rehabilitation practices upon evidence.

**Method:**

Supported by a committee of stakeholders, we will search electronic databases for research articles examining the association between length of stay or intensity of inpatient rehabilitation services and outcomes or the determinants of inpatient rehabilitation length of stay in adults with neurotrauma published after January 1990. Two researchers will independently screen the article titles and abstracts for inclusion. Two reviewers will independently extract the data. Primary outcomes of interest will be level of function, participation and return to work. If the data allow it, a meta-analysis of the studies will be performed.

**Discussion:**

The results of this systematic review will clarify the factors that influence length of stay and intensity of rehabilitation services for individuals with TBI and SCI. They will give clinicians indications for optimal length of stay in these patient populations, contributing to better quality of care and better functional results.

**Trial registration:**

This review protocol has been registered on the PROSPERO database (CRD42012003120) and is available at http://www.crd.york.ac.uk/PROSPERO/display_record.asp?ID=CRD42012003120.

## Background

Injuries are one of the leading causes of disabilities around the world [1]. Neurotraumas (that is, traumatic brain injury (TBI) and spinal cord injury (SCI)) are especially devastating since they often affect young people, create permanent neurological damage and, by their very nature, affect multiple organic systems (neurological, muscle, cognitive, and so on). Individuals with TBI or SCI must suddenly deal with physical, cognitive, behavioral and emotional impairments that have the potential to create serious disabilities, especially in regard to their work, productivity, and social and family responsibilities [2,3]. Rehabilitation interventions are a key component of the services required by individuals with neurotrauma to recover or compensate for impaired abilities and achieve optimal social participation.

At the present time, there is some evidence of the benefit of rehabilitation for neurotrauma populations [4,5]. Indeed, it is recognized that after a neurotrauma, rehabilitation services reduce disability and improve function, allowing trauma survivors to recover some participation in their activities. However, it is also recognized that some recovery will occur without professional support, sometimes stimulated by individuals’ involvement in their usual activities in their natural setting. Rehabilitation professionals thus face a difficult decision regarding whether or not a patient could benefit from more professional services and consequently whether or not they should end rehabilitation services. Currently, little is known about the optimal ‘dose’ of rehabilitation required by these individuals, for example the number of hours or days of rehabilitation likely to produce a better functional result. There is no consensus concerning the optimal intensity of services or length of stay for people with TBI or SCI. Consequently, the length of stay and intensity of rehabilitation services vary significantly between providers and facilities [6] and clinicians are left without clear indications regarding the optimal dose of rehabilitation to provide to these populations. This situation is critical. On the one hand, less than optimal service provision could limit individuals’ abilities and participation and generate considerable social costs, such as support services or replacement income. On the other hand, if services exceed the optimal breakpoint, some of the care will have a reduced impact on the person’s functional status. Offering such maximum rehabilitation services would be costly, not just financially but in terms of lost opportunities since the extra services received by the person with neurotrauma could not be offered to other clients on a waiting list or with different needs.

In the current context of labor shortages and financial restrictions, it is essential to give clinicians and managers clear indications about the optimal dose of rehabilitation services in order to improve the efficiency of neurotraumatology rehabilitation services.

Many primary studies have produced mixed evidence of the effect of rehabilitation length of stay on individuals with neurotrauma [7-13]. However, to date no systematic review of this evidence has been performed. This makes it difficult for managers and clinicians to base their rehabilitation practices upon evidence. The Knowledge to Action framework [14] suggests that a systematic review and synthesis of existing primary studies should be carried out before a knowledge translation tool can adapt this evidence into actionable and easily implementable recommendations.

### The goals of this research are

1. To systematically review the effect of the length of stay and rehabilitation intensity on outcomes in terms of functional independence and social participation of individuals with TBI or SCI;

2. To explore how fixed factors (for example age, trauma severity) and variable factors (for example presence of co-morbidity) influence the rehabilitation length of stay of individuals with TBI or SCI.

## Methods

This systematic review protocol was developed using guidance from the Cochrane Collaboration [15]. The review protocol was registered with the PROSPERO International Prospective Register of Systematic Reviews [16] (Registration number CRD42012003120) to reduce the variability in the review process and increase the validity of the results [15,17]. To increase the impact of our review [18], a committee of stakeholders (neurotrauma rehabilitation program clinicians, users, managers) has been asked to assist the review process by collaborating during specific steps.

### Literature search

We will search electronic databases using a strategy developed by the researchers using the medical subject headings pertaining to each database. The stakeholders’ committee will examine the search strategy for its comprehensiveness (key words and data sources). The quality of this strategy will be assessed by at least two independent information specialists using the Peer Review of Electronic Search Strategies Evidence-Based Checklist (PRESS EBC) [19,20].

A professional librarian will perform the primary search in nine electronic databases: PubMed, EMBASE, CINAHL, AMED, PsychINFO, the Cochrane Library, FirstSearch, Web of Science, ProQuest dissertations and theses to retrieve primary studies published from January 1990 to October 2012. A secondary search will be performed by checking the reference lists of the studies included in the review. Unpublished, non-peer-reviewed sources (gray literature) will also be searched. For example, we will explore websites and publications of organizations dedicated to individuals with neurotrauma, official agencies, Google, and so on.

### Study selection

Studies examining 1) the association between length of stay or intensity of inpatient rehabilitation services and outcomes (level of function, participation, return to work), or 2) the determinants of inpatient rehabilitation length of stay in adults with neurotrauma (moderate and/or severe TBI or SCI), and 3) published after January 1990 will be considered. In a first selection phase, two review authors (CG, MEL) will independently screen the title and abstract of all identified studies for inclusion against the eligibility criteria.

#### Inclusion criteria

•Languages: English and French.

•Publication types: experimental and epidemiological study designs including randomized control trials, non-randomized controlled trials, quasi-experimental, before and after studies, prospective and retrospective cohort studies, case control studies and analytical cross-sectional studies. Gray literature, qualitative and observational studies will also be taken into consideration.

•Study population: adult (18 to 65 years old without geriatric profile) with moderate or severe traumatic SCI, adult with moderate or severe TBI.

•Intervention setting: inpatient rehabilitation.

#### Exclusion criteria

•Publication types: editorial, case report, anecdotal report.

•Study population: non-traumatic injuries (for example cancer, stroke, poliomyelitis, and so on), alternate level of care, children only, older people only.

•Intervention setting: acute rehabilitation, outpatient rehabilitation, and community integration setting.

In a second selection phase, two independent reviewers (MEL, CG) will confirm the eligibility of the studies by reviewing the full text of the selected studies. A third party (LN) will consider all discrepancies not resolved by discussion in the course of the two selection phases and make a final decision about eligibility. A kappa statistic and the percent agreement will be used to calculate interrater agreement in both selection phases.

The stakeholders’ committee will be informed of the progress of study selection and invited to complete the list of selected studies by suggesting additional studies or results from gray literature.

### Data extraction

Two reviewers (MEL, ASA) will independently extract data from the studies regarding 1) general information, 2) study characteristics, 3) participants’ characteristics, 4) outcomes, and 5) results, using a standardized form. A first extraction will be performed on 10 articles to pilot the extraction form and insure the comprehensiveness and reliability of the data extraction. The results of this pilot extraction will be examined to refine the process. Disagreements between the reviewers will be resolved by discussion and consensus. If a consensus cannot be reached, a third researcher will make the final decision (CG).

### Risk of bias assessment

The Cochrane Collaboration's tool for assessment of risk of bias will be used to assess the risk of bias of randomized studies. The Transparent Reporting of Evaluations with Nonrandomized Design (TREND) grid [21] will be used to assess risks of bias in non-randomized studies. A risk of bias table will be generated with the principal biases and the methodological quality of the studies.

### Data analysis

A narrative synthesis method [22] will first be used to describe the results of the systematic review. All included studies will be summarized in narrative form, and summary tables will be created showing key study characteristics (population characteristics, study outcomes, sample sizes, settings, statistics used, related results, and any other important aspect related to each research question of interest).

Then, when possible, meta-analysis methods will be applied [23]. The tau-squared and I^2^ statistic will be used to quantify the statistical heterogeneity between studies examining our outcomes of interest, where *P* <0.10 and where I^2^ is larger than 50%, indicating a high level of statistical heterogeneity between the studies. If characteristics of the studies are homogeneous enough, we will group them and perform meta-analysis of the pooled data. If outcomes of interest of each included study were reported using different outcome measures on a continuous scale, the summary measures of effect in the form of standardized mean difference (SMD) for each outcome will be generated. All analyses will be conducted with R software (available at http://www.r-project.org).

We will ask two independent experts to assess the quality of our systematic review using the Preferred Reporting Items for Systematic Reviews and Meta-Analyses (PRISMA) tool [24,25] and its development and interpretation guide [24].

The partners of the stakeholders’ committee will receive a synthesis of the narrative synthesis and meta-analysis results, to serve as a basis for formal discussion in a one-day meeting. This knowledge exchange activity will provide guidance for the interpretation of the results of the systematic review [26]. It will increase our understanding of the impacts of the length of stay on actual rehabilitation practice and hopefully facilitate knowledge dissemination and appropriation by the principal clinical users.

## Discussion

The results of this systematic review will clarify the factors that influence length of stay and intensity of rehabilitation services for individuals with TBI and SCI. More importantly, the results will give clinicians indications for optimal length of stay in these patient populations, contributing to better quality of care and better functional results. The partners of the collaborative committee will be able to identify the clients who are at risk for an increased length of stay or who require more intensive provision of rehabilitation services. They will be able to better understand their administrative data and evaluate their service against evidence-based criteria. The results of this systematic review could be included in a clinical guideline or be used to develop performance indicators for organizations offering services to individuals with TBI or SCI.

## Abbreviations

SCI: Spinal cord injury; SMD: Standardized mean difference; TBI: Traumatic brain injury

## Competing interests

The authors declare that they have no competing interests.

## Authors’ contributions

MEL conceived and designed the review and took the lead in writing this manuscript. CG supported the conception of the review, obtained funding and participated in the manuscript writing. ASA participated in the review conception and drafted the manuscript. LN supported the conception and design of the study and participated in writing the manuscript. All authors approved the final manuscript.

## Authors’ information

Complete departmental affiliations: Marie-Eve Lamontagne, Center for Interdisciplinary Research in Rehabilitation and Social Integration, Institut de réadaptation en déficience physique de Québec, QC, Canada, Department of Rehabilitation, Université Laval, Québec, QC, Canada;

Cynthia Gagnon, Faculty of Medicine and Health Sciences, Université de Sherbrooke, Québec, QC, Canada; Groupe de recherche interdisciplinaire sur les maladies neuromusculaires (GRIMN), Neuromuscular Clinic, Centre de réadaptation en déficience physique de Jonquière, Centre de santé et de services sociaux de Jonquière, Québec, QC, Canada;

Anne-Sophie Allaire, Center for Interdisciplinary Research in Rehabilitation and Social Integration, Institut de réadaptation en déficience physique de Québec, QC, Canada;

Luc Noreau, Center for Interdisciplinary Research in Rehabilitation and Social Integration, Institut de réadaptation en déficience physique de Québec, QC, Canada, Department of Rehabilitation, Université Laval, Québec, QC, Canada.

## References

[B1] World Health Organization The global burden of disease: 2004 update The global burden of disease 2004 Geneva: World Health Organization 146 2008:146

[B2] Klonoff PS Lamb DG Henderson SW Outcomes from milieu-based neurorehabilitation at up to 11 years post-discharge Brain Inj 2001 15 413 428 11350655 10.1080/02699050010005968

[B3] Malec JF Smigielski JS De Pompolo RW Thompson JM Outcome evaluation and prediction in a comprehensive-integrated post-acute outpatient brain injury rehabilitation programme Brain Inj 1993 7 15 29 10.3109/02699059309008153 8425113

[B4] Cullen N Chundamala J Bayley M Jutai J The efficacy of acquired brain injury rehabilitation Brain Inj 2007 21 113 132 10.1080/02699050701201540 17364528

[B5] NIH Rehabilitation of persons with traumatic brain injury NIH Consens Statement 1998 16 1 41 10874909

[B6] Zhu XL Poon WS Chan CH Chan SH Does intensive rehabilitation improve the functional outcome of patients with traumatic brain injury? Interim result of a randomized controlled trial Br J Neurosurg 2001 15 464 473 10.1080/02688690120097688 11813997

[B7] Blackerby WF Intensity of rehabilitation and length of stay Brain Inj 1990 4 167 173 10.3109/02699059009026162 2331546

[B8] Spettell CM Ellis DW Ross SE Sandel ME O'Malley KF Stein SC Spivack G Hurley KE Time of rehabilitation admission and severity of trauma: effect on brain injury outcome Arch Phys Med Rehabil 1991 72 320 325 2009049

[B9] Prigatano G Wong J Williams C Plenge K Prescribed versus actual length of stay and inpatient neurorehabilitation outcome for brain dysfunctional patients Arch Phys Med Rehabil 1997 78 621 629 10.1016/S0003-9993(97)90428-7 9196470

[B10] Stonnington HH Length and intensity of rehabilitative involvement Brain Inj 2000 14 393 395 10.1080/026990500120501 10834335

[B11] Rehab center cuts LOS with interdisciplinary teams Hosp Case Manag 1997 5 69 72 10166646

[B12] Al-Jadid M Robert AA An analysis of the length of stay in traumatic and non-traumatic spinal cord injured patients. A rehabilitation unit experience in Saudi Arabia Saudi Med J 2010 31 555 559 20464047

[B13] Burns AS Yee J Flett HM Guy K Cournoyea N Impact of benchmarking and clinical decision making tools on rehabilitation length of stay following spinal cord injury Spinal Cord 2013 51 165 169 10.1038/sc.2012.91 22847654

[B14] Graham ID Logan J Harrison MB Straus SE Tetroe J Caswell W Robinson N Lost in knowledge translation: time for a map? J Contin Educ Health Prof 2006 26 13 24 10.1002/chp.47 16557505

[B15] Higgins JPT, Green S Cochrane handbook for systematic reviews of interventions Version 5.1.0 [updated March 2011], The Cochrane Collaboration 2011 Available from http://www.cochrane-handbook.org

[B16] PROSPERO international prospective register of systematic reviewshttp://www.crd.york.ac.uk/prospero/10.1186/2046-4053-1-2 PMC3348673 22587842

[B17] Straus S Moher D Registering systematic reviews Can Med Assoc J 2010 182 13 14 10.1503/cmaj.081849 19620270 PMC2802597

[B18] Keown K Van Eerd D Irvin E Stakeholder engagement opportunities in systematic reviews: knowledge transfer for policy and practice J Contin Educ Health Prof 2008 28 67 72 10.1002/chp.159 18521874

[B19] McGowan J Sampson M Lefebvre C An evidence based checklist for the peer review of electronic search strategies (PRESS EBC) Evid Base Libr Info Prac 2010 5 149 154

[B20] Sampson M McGowan J Cogo E Grimshaw J Moher D Lefebvre C An evidence-based practice guideline for the peer review of electronic search strategies J Clin Epidemiol 2009 62 944 952 10.1016/j.jclinepi.2008.10.012 19230612

[B21] Des Jarlais DC Lyles C Crepaz N Improving the reporting quality of nonrandomized evaluations of behavioral and public health interventions: the TREND statement Am J Public Health 2004 94 361 366 10.2105/AJPH.94.3.361 14998794 PMC1448256

[B22] Schünemann HJ Oxman AD Higgins JP Vist GE Glasziou P Guyatt GH Higgins JPT, Green S Chapter 11: Presenting results and “Summary of Findings” tables Cochrane handbook for systematic reviews of interventions 2011 Chichester, West Sussex: Hoboken NJ: John Wiley & Sons 335 338

[B23] Deeks JJ Higgins J Altman DG Chapter 9: Analysing data and undertaking meta-analyses Cochrane handbook for systematic reviews of interventions 2011 Chichester, West Sussex: Hoboken NJ: John Wiley & Sons 234 296

[B24] Liberati A Altman DG Tetzlaff J Mulrow C Gøtzsche PC Ioannidis JPA Clarke M Devereaux PJ Kleijnen J Moher D The PRISMA statement for reporting systematic reviews and meta-analyses of studies that evaluate health care interventions: explanation and elaboration PLoS Med 2009 6 e1000100 10.1371/journal.pmed.1000100 19621070 PMC2707010

[B25] Moher D Liberati A Tetzlaff J Altman DG The PG Preferred reporting items for systematic reviews and meta-analyses: the PRISMA statement PLoS Med 2009 6 e1000097 10.1371/journal.pmed.1000097 19621072 PMC2707599

[B26] Tricco AC Straus SE Moher D How can we improve the interpretation of systematic reviews? BMC Med 2011 9 31 10.1186/1741-7015-9-31 21450084 PMC3072347

